# Utility of Post-ureteroscopy Lesion Scale (PULS) in Per-operative Decision-Making for the Need of Double J Stent

**DOI:** 10.7759/cureus.26166

**Published:** 2022-06-21

**Authors:** Arsalan Pervaiz, Wajahat Aziz, M Hammad Ather

**Affiliations:** 1 Section of Urology, Department of Surgery, Aga Khan University, Karachi, PAK

**Keywords:** validation, ureteric injury, dj stenting, ureterorenoscopy, puls score

## Abstract

Objectives: To assess the applicability of the post ureteroscopy lesion scale (PULS) as an objective measure to define the need for double J (DJ) stent placement after ureterorenoscopy (URS).

Methods: Between June and December 2020 a cross-sectional study was conducted at a university hospital. All patients fulfilling the inclusion criteria undergoing URS for renal or ureteric stones were included. At the completion of procedures, the ureter was carefully inspected for injury. Lesions were classified using PULS scoring by the operating surgeon, another consultant, and the resident. The primary outcome was to validate the PULS score against the surgeon’s decision for postoperative stenting and to assess its reliability.

Results: A total of 126 patients were included with a mean age of 43.42±15.3 years. The mean stone size was 9.42±3.60mm. DJ stents were placed in 81 cases (62.4%). All of the 38 (30.1%) patients with a significant residual fragment were stented. Ureteric injury of grade 1 was observed in 66 patients (52.3%), of which 22 (33%) had DJ stenting. PULS grade 2 injuries were observed in 22 patients (17.4%), and 95% were stented. With a PULS score of > 2 almost all (97.8%) were stented. Inter-rater reliability of PULS scoring was high among the consultants (Kendall’s W=0.89, p<0.005).

Conclusion: DJ stent placement was observed in 33%, 95%, and 98% of patients with PULS grade 1, 2, and >2 injury respectively. In patients with no residual fragment, the need for DJ stenting can be objectively defined using the PULS scoring system as it has high specificity and good interrater reliability.

## Introduction

Upper tract urolithiasis is a highly prevalent disease worldwide, with rates ranging from 7 to 13% in North America, 5-9% in Europe, and 1-5% in Asia [1]. Stones that fail to pass spontaneously or by assisted medical treatment require an endourological procedure. Double J (DJ) stents are commonly placed for effective drainage of the urinary tract following ureterorenoscopy (URS). DJ stents minimize the risk of postoperative obstruction from edema and promote ureteric healing [2]. However, DJ stents are frequently associated with bothersome side effects and impacts patient quality of life [3]. These stent-related symptoms often require medical treatment with a variable success rate [4]. However, an ideal approach would be to avoid DJ stenting whenever possible. European and American association of urology guidelines also suggest that after uncomplicated ureterorenoscopy DJ stenting can be safely omitted [5]. In the contemporary literature, there is a dearth of clear objective criteria to define uncomplicated ureteroscopy [6]. There are some imperative indications of stenting following ureteroscopy including single kidney, residual stones, impacted stones, ureteral wall edema, and per operative ureteral injury.

One of the ways of defining uncomplicated ureteroscopy is to utilize an objective scoring system like the post ureteroscopy lesion scale (PULS) first reported by Schoenthaler et al. in 2012 [7]. PULS enables standardization of the description of iatrogenic ureteral lesions during ureterorenoscopy and has the potential to objectively define the need for postoperative DJ stenting [8].

DJ stent placement after URS is often required. Post URS ureteric trauma is an important factor in deciding the need for DJ stent placement. In the current practice stenting after URS is a subjective decision. PULS is a standardized way of describing iatrogenic ureteral lesions during URS and can be used to objectively define the need for postoperative DJ stenting.

## Materials and methods

Between June 2020 and December 2020, 126 consecutive adult (>18 years) patients undergoing elective ureterorenoscopy (URS) at the Urology Department of a University Hospital were included. Patients with pre-URS stenting, solitary kidney, active infections, or ancillary procedures with URS were excluded. Also patients with a history of ureteric strictures or open surgery requiring DJ stenting were also excluded.

After Aga Khan University Hospital Ethics Review Committee issued approval 2020-4791-10900, data were collected prospectively by chart review for patient demographic details, comorbidities, American Society of Anesthesiology score (ASA), stone-related factors, and pain score on a visual analog scale at the time of discharge. Consultant urologists with experience in at least 50 independent URS performed the procedure. URS was performed either using semi-rigid or rigid ureteroscope or Cobra dual-channel flexible ureterorenoscope (Richard Wolf™) or WiScope® Single-Use Digital Flexible Ureteroscope. Stones were fragmented using pneumatic lithotripter, SWISS lithoclast™ (distal ureteral stones) Holmium-YAG, Lumenis™ 100W, (middle, proximal ureteral and renal stones).

At the completion of the URS the entire length of the ureter was inspected from the pelvi-ureteric junction to the ureteric orifice. Injuries were rated according to the PULS grading system. The operating surgeon, another consultant and the residents were asked to grade the injury independently. Post URS stenting status was recorded and the operating surgeon documented the indication and planned duration of stenting. Patients with residual stones were excluded from the final analysis. Complications were noted on follow-up visits within 30 days. Readmission and ER visits within 30 days were noted from hospital record. Stone clearance was noted on postoperative x-ray, ultrasound, or computed tomography of kidneys, ureters and bladder (CT KUB) at the discretion of the admitting urologist.

Statistical analysis was performed using the Statistical Package for Social Sciences (SPSS) version 23 (IBM Corp., Armonk, NY, USA). Continuous variables were described in terms of mean ± SD while qualitative variables were described using frequency and percentages. Multivariate analysis was conducted for factors associated with stenting. Reliability of the tool was reported by Kendall tau statistics by assessing agreement between the two raters. Sensitivity specificity, positive predictive value (PPV), and negative predictive value (NPV) of PULS were calculated by using standard formula considering surgeons' decision as a gold standard. A p-value <0.05 was considered significant throughout the study.

## Results

This study included 126 patients who underwent ureterorenoscopy from June to December 2020. Two-thirds (67.46%) were male; the mean age was 43.42±15.3 years. The mean stone size was 9.3±3.60mm. Approximately half (49.2%) of the stones were upper ureteric or renal. Most (81%) of the patients underwent semi-rigid URS. Flexible URS was done in the remaining patients (19%). One in three patients required ureteric dilatation, using reusable metal dilators, and access sheath was used in 21% (Table 1).

**Table 1:** Patients demographic and stone related characteristics

	TOTAL (126)	STENTED GROUP 81	UNSTENTED GROUP 45	P<0.05
AGE (years)	43.42 ± 15.3	41.59±14.46	46.7± 16.57	0.07
GENDER				
MALE	85	51	34	0.15
FEMALE	41	30	11	
BMI (kg/m^2^)	27.1± 4.82	27.4± 5.14	26.6± 4.21	0.37
DIABETES	32	22	10	0.54
SIDE				
LEFT	69	43	26	0.612
RIGHT	57	38	19	
STONE PARAMETERS				
STONE SIZE (mm)	9.43+3.61	10.12+3.39	8.20+3.76	0.004
SITE (KIDNEY /UPPER URETER)	61	34	27	0.064
SITE (LOWER/MID URETER)	65	47	18	
HYDRONEPHROSIS (mm)	102	70	32	0.036
PAIN SCORE (VAS)	2.19+0.92	2.23 +0.91	2.11+0.96	0.47
30 DAY READMISSION	3	1	2	0.712

DJ stents were placed in 81 cases (64.2%). In most of these cases (47/81) stent was placed for a one- to four-week period. Ureteric injury of grade 1 or less was seen in 99 patients (78.6%) and in 55 of them, a DJ stent was placed. Ureteric injury of grade 2 was observed in 27 patients (21.4%), and all but one of these patients had a DJ stent placed. No grade 3 or above injuries were observed in our series. Thirty-eight patients had residual stones and a DJ stent was placed in all of them and these were excluded from the final analysis for calculation of sensitivity and specificity. Three patients were readmitted within 30 days (two with ureteric colic and one with UTI), and none of the patients had Clavien grade 3 or above complications. Outcome variables like pain score and 30-day readmissions were similar in both groups (Table 1).

A PULS score of ≥ 2 was found to be quite specific (97.8 %) for decision regarding DJ stenting but had low sensitivity (48.8%) (Table 2).

**Table 2:** Stent placement in various grades of PULS injury scale, with sensitivity, specificity, NPV and PPV of PULS score and stenting PULS: post ureteroscopy lesion scale, NPV: negative predictive value, PPV: positive predictive value

			VALUE	95% CI
	PULS 0,1	PULS 2,3	SENSITIVITY	48.84%	33.31% TO 64.54%
UNSTENTED GROUP (N=45)	44	1	SPECIFICITY	97.78%	88.23% TO 99.94%
STENTED GROUP (N=43)	22	21	POSITIVE PREDICTIVE VALUE	95.45%	74.70% TO 99.34%
			NEGATIVE PREDICTIVE VALUE	66.67%	59.82% TO 72.88%
			ACCURACY	73.86%	63.41% TO 82.66%

**Figure 1:**
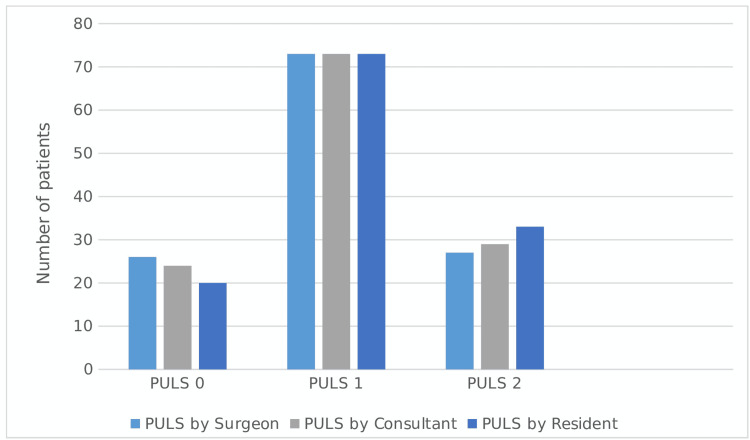
Inter observer variability in post ureteroscopy lesion scale score (PULS) between primary operating surgeon, other consultant urological surgeon and the scrubbed resident

Inter-rater reliability was high both among consultants (Kendall W=0.92; Spearman’s 0.93) and also between residents and the consultant (Kendall’s W =0.89; Spearman’s 0.89) (Figure 1).

Stone size was significantly larger in patients who had DJ stenting (10.12±3.39 vs. 8.20±3.76; p value=0.004). Moreover patients with hydronephrosis required DJ stenting more frequently (70/81 vs. 32/45; p=0.036). Similarly, patients requiring ureteric dilatation also required DJ stent more frequently (35/81 vs. 7/45; p=0.002). However, on multivariate regression analysis, only stone size and ureteric dilatation were found to be significant predictors of DJ stenting.

## Discussion

URS remains the commonest surgical intervention in the management of ureteral stones [9]. Endourological interventions for the treatment of a ureteric stone are often accompanied by the placement of DJ stents. The most frequent indication for ureteric stenting is the drainage of the upper tract and to decrease pain from ureteral wall edema and stone fragmentation following URS.

URS is known to result in some degree of ureteral trauma. Post URS complication rates of 9-11% have been reported, with ureteric perforation accounting for 1-4% [10-14]. Ureteric trauma of varying degrees following URS is an important predictor of DJ stenting. However, indication for post-URS stenting remains largely subjective. European Association of Urology (EAU) and American Urological Association (AUA) guidelines states that it is optional to place an indwelling ureteral stent post uncomplicated ureteroscopy [15,16]. The definition of uncomplicated ureteroscopy too remains subjective. Attempts have been made to assess the use of an injury grading system in helping with this dilemma. We have used the PUL Scale to delineate the degrees of ureteric injury and its applicability for the decision regarding stenting. 

Generally the operating surgeon gravitates more towards stenting, resulting in high tendency (63-80%) towards stenting following URS [17,18]. Over 90% of the urologists in a US-based survey were in favor of stenting even after an uncomplicated URS [19]. Stents were placed in most of the cases in our study. Similar findings have been reported in a multi-institutional study, where 65% of the patients had postoperative stenting [20]. Another prospective audit from eight centers in the United Kingdom showed around 74% of patients had some form of ureteric drainage following URS with 68% having stents [18].

Decision to stent or not to stent can be predicted by several patient- and procedural-related factors. The findings of CORES URS Global study suggested the need for an individualized postoperative stenting strategy [21]. Limited insight is available in literature on the factors predicting the necessity of postoperative stenting. Our data highlighted the presence of preoperative hydronephrosis and dilation of the ureteric orifice as the factors associated with increased need for post-URS stenting. This could be explained by the ureteric wall edema resulting from dilatation of the ureter. Boddy et al. reported in an animal study that ureteric edema and upper tract obstruction on imaging lasted for at least 96 hours after ureteric dilation, however there are no equivalent studies in humans [22]. A survey among US-based urologists identified ureteral edema in 77% of the cases as the reason for placement of stents after uncomplicated URS [20]. Postoperative stenting in our study was mainly performed for residual stone, trauma and reasons related to ureteral anatomy i.e. ureteric kink, tight ureter and narrow ureteric orifice.

PULS showed a good specificity for postoperative stenting when compared with the surgeon’s decision but had low sensitivity. After application of the PUL Scale, a grade 0-1 lesion was seen in the majority of our patients. DJ stent was placed even in patients with low-grade injury (≤1PULS) without any residual stones. We feel that the DJ stent could have been safely omitted in these patients.

Considering the adverse effects on the quality of life [23,24], stent placement should ideally be omitted after uncomplicated URS and where required should preferably be kept for the shortest duration necessary. Open-end catheter secured to a Foley catheter overnight is described as a cost-effective measure, obviating stent-related symptoms as well [25]. There is no consensus on the stent indwelling time after URS. Although stent dwell times (more than four weeks) are not beneficial and can even be harmful, it is still to be established whether a shorter stent dwell time can reduce patient morbidity [26]. Nikita et al. reported an ideal duration of dwell time of five days [20]. However, in our practice most of the surgeons preferred to keep stents for one to four weeks.

Our study has certain limitations. It is a single center project, and the analysis does not include all information relevant to postoperative stent placement (stent-related symptoms, stone analysis). Despite these factors, we believe this evidence provides a basis for devising a strategy that is individualized for postoperative stenting in endourology.

Validation of the PULS scoring system will provide clinicians with a tool that can reliably predict the need for Double J stenting and will help clinicians in decision-making and guiding patients about the most appropriate treatment option. After the implementation of PULS-based stenting in clinical practice the number of patients getting unnecessary stenting after uncomplicated URS would be limited. This would be cost-effective as well and the complications associated with stenting would be avoided. Furthermore, audit of postoperative complications including pain score and ER visits after implementation of PULS-based DJ stenting is desirable to validate our results.

## Conclusions

DJ stents are frequently placed following URS. Besides residual stones, ureteric trauma is a major reason for placement of DJ stents. In patients without significant residual stones, an objective evaluation of ureteral wall injury provides a rational basis for indicating stents. PULS score shows a high sensitivity and inter-rater reliability and can be easily used for objectively defining uncomplicated ureterorenoscopy and decision for post-URS DJ stenting.
